# Effects of palonosetron for prophylaxis of postoperative nausea and vomiting in high-risk patients undergoing total knee arthroplasty: A prospective, randomized, double-blind, placebo-controlled study

**DOI:** 10.1371/journal.pone.0196388

**Published:** 2018-05-14

**Authors:** Jung-Hee Ryu, Young-Tae Jeon, Byunghun Min, Jin-Young Hwang, Hye-Min Sohn

**Affiliations:** 1 Department of Anesthesiology and Pain Medicine, Seoul National University Bundang Hospital, Seongnam, Republic of Korea; 2 Department of Anesthesiology and Pain Medicine, College of Medicine, Seoul National University, Seoul, Republic of Korea; 3 Department of Anesthesiology and Pain Medicine, SMG-SNU Boramae Medical Center, Seoul, Republic of Korea; University of Pittsburgh, UNITED STATES

## Abstract

**Background:**

The preemptive multimodal pain protocols used in total knee arthroplasty (TKA) often cause emesis postoperatively. We investigated whether palonosetron prophylaxis reduces postoperative nausea and vomiting (PONV) in high-risk patients after TKA.

**Methods:**

We randomized 120 female patients undergoing TKA to receive either palonosetron (0.075 mg, intravenous) or no antiemetic prophylaxis (0.9% saline, control group). All patients were given spinal anesthesia, a continuous femoral nerve block, and fentanyl-based intravenous patient controlled analgesia. Patients undergoing staged bilateral TKA were assigned to one group for the first knee and the other group for the second knee. The overall incidence of PONV, the incidences of both nausea and vomiting, severity of nausea, complete response, requirement for rescue antiemetics, pain level, opioid consumption, and satisfaction scores were evaluated during three periods: 0–2, 2–24, and 24–48 h postoperatively. We also compared PONV and pain between the first and second TKA.

**Results:**

The incidence of PONV during the first 48 h was lower in the palonosetron group compared with the controls (22 *vs*. 41%, *p* = 0.028), especially 2–24 h after surgery, as was the nausea and vomiting respectively. The severity of nausea was lower in the palonosetron group (*p* = 0.010). The complete response rate (93 *vs*. 73%, *p* = 0.016) and satisfaction score (84 ± 12 *vs*. 79 ± 15, *p* = 0.032) were higher in the palonosetron group during 2–24 h after surgery. Patients who underwent a second operation complained of more severe pain, and consumed more opioids than those of the first operation. There was no difference in the incidence of PONV between the first and second operations.

**Conclusions:**

Palonosetron prophylaxis reduced the incidence and severity of PONV in high-risk patients managed with multimodal pain protocol for 48 h, notably 2–24 h after TKA.

## Introduction

The incidence of postoperative nausea and vomiting (PONV) in patients undergoing arthroplasty ranges from 68% to 83% in patients not given prophylactic antiemetics [1]. The multimodal pain protocols, using more than one analgesic with different mechanisms due to the limited effects of single-drug therapy [2,3], are widely used to relieve pain after total knee arthroplasty (TKA). The multimodal pain approach results in better pain control [4] and lower side effects, but still provokes more emetic events than traditional pain control [5,6]. Various methods are recommended to decrease PONV, including prophylactic antiemetics, regional anesthesia, propofol, oxygen, fluid infusion, minimal opioid use, and corticosteroids [1,7]. Despite these preventive methods, the incidence of PONV (40%) after TKA is still high [8].

Palonosetron, the latest 5-HT_3_ receptor antagonist, has a longer half-life and greater receptor affinity than other antagonists [9,10]. Palonosetron is effective against nausea and vomiting in patients using anticancer drugs [11] and for preventing PONV [12]. However, the antiemetic efficacy of palonosetron prophylaxis in high-risk patients managed with multimodal analgesic regimens and had at least two risk factors of PONV, remains unclear [9,13].

Pain is increased in patients undergoing staged TKA, in whom the second operated knee has greater sensitivity as a result of the surgical injury to the first operated knee, and more analgesics are required [14]. The effect of antiemetics on PONV has not been evaluated in patients undergoing the second surgery in staged bilateral TKA.

This prospective, randomized, double-blinded, case-controlled trial investigated the antiemetic efficacy of palonosetron for the prevention of PONV in high-risk patients receiving multimodal analgesics including fentanyl-based patient-controlled analgesia (PCA) following TKA under spinal anesthesia.

## Materials and methods

### Patient population

This study was approved by the institutional review board of Seoul National University Bundang Hospital and registered at the Clinical Research Information Service (cris.nih.go.kr, registration number KCT0000485). It was conducted according to the revised Declaration of Helsinki of the World Medical Association and ICH GCP guidelines for good clinical trial practice.

We studied 120 patients undergoing elective TKA after obtaining written informed consents. Inclusion criteria were as follows; female patients aged 18–85 years, scheduled for unilateral or staged bilateral TKA for primary osteoarthritis under spinal anesthesia, and postoperative pain management using intravenous PCA and continuous femoral nerve block (FNB), and ASA physical status I or II, and at least two additional risk factors: history of motion sickness or PONV, non-smoking status, and use of opioids for postoperative pain [15]. Exclusion criteria were patients undergoing revision TKA, simultaneous bilateral TKA, or general or epidural anesthesia, patients who took opioids or steroids within 1 week of surgery, antiemetic medication within 1 day of surgery, renal insufficiency (serum Cr > 1.6 mg/dl), were unable to use intravenous PCA or continuous FNB, were unable to understand the numeric rating scale (NRS) for pain or express the degree of PONV, abused alcohol or drugs, or had diseases of the digestive system.

Block randomization with a block size of four was used based on a computer-generated schedule. The group assignments were sealed in sequentially numbered, opaque envelopes. Patients undergoing unilateral TKA were assigned to either the palonosetron or control group. Patients undergoing staged bilateral TKA, with a 1-week interval between stages 1 and 2, were randomly assigned to one group according to the first knee. One-week later, in the second knee operation, the group assignment was inevitably determined to be the other remaining group. In other words, the second knee could not be a blinded assignment and was automatically determined.

### Routine pre- and postoperative care and data collection

All patients were given the same anesthetics and multimodal pain protocol, except palonosetron. Preoperative preemptive analgesics using oral acetaminophen 650 mg, celecoxib 200 mg, pregabalin 75 mg and intravenous dexamethasone 10 mg were administered on the ward on a call basis. On arrival at the surgical reception area, patients were premedicated with midazolam 0.03 mg/kg intravenously and a continuous FNB was established (0.2% ropivacaine solution at 5 mL/h, no bolus, total 250 mL) with ultrasound-guided technique. Spinal anesthesia consisted of bupivacaine 10 to 15 mg and adjuvant fentanyl 15 to 20 μg. A continuous propofol infusion was used to induce sedation during surgery. Oxygen 5 L/min was supplied by facial mask. Adequate spontaneous breathing was confirmed with EtCO_2_ monitoring.

All surgeries were conducted by one of two surgeons using a standard medial parapatellar arthrotomy with a tourniquet. A posterior-stabilized prosthesis (Genesis II; Smith & Nephew, Memphis, TN, USA) with a fixed-bearing system was used in all TKAs. A drug cocktail consisting of morphine 5 mg, ropivacaine 300 mg, and ketorolac 30 mg was injected periarticularly after all prostheses had been inserted.

Thirty minutes before the end of surgery, either palonosetron (palonosetron group, *n* = 60) or saline (control group, *n* = 60) was injected according to the group assignment, and intravenous PCA was started. No additional antiemetics were mixed with the PCA formulation. In the palonosetron group, 0.075 mg (1.5 ml) palonosetron was injected as a bolus, and 1.5 ml normal saline was injected in the control group. The patients and anesthesia providers were blinded to the group assignments; a trained nurse who was not involved in the study prepared the drugs in identical syringes. The PCA formulation was programmed to deliver 1 ml of a 100-ml solution containing 1,500 μg fentanyl for patients who were 60 years or younger or 1,200 μg for patients older than 60 years when the button was pressed, with a 10-minute lockout period without a basal infusion. Once the patients resumed oral intake, acetaminophen 650 mg, celecoxib 200 mg, and pregabalin 75 mg were administered every 12 h. Intramuscular ketoprofen (100 mg) was used for acute pain rescue and intravenous metoclopramide (10 mg) was administered for PONV.

Another anesthesiologist blinded to the group allocations evaluated the patients 0–2, 2–24 and 24–48 h after surgery, in terms of the overall incidence of PONV, the respective incidences of both nausea and vomiting, severity of nausea, complete response to antiemetics, severity of pain, amount of PCA fentanyl used, need for additional antiemetics and analgesics, side-effects, and satisfaction score.

We performed subgroup analyses to assess whether previous surgical injury affects pain and PONV in patients who had staged sequential TKA. We compared PONV-related variables, rescue antiemetics, postoperative pain intensity, and opioid consumption in patients undergoing staged bilateral TKA according to the order of surgery.

The primary outcome variables were the incidence of PONV during the first 48 h postoperatively. Nausea was defined as a subjective unpleasant feeling associated with awareness of the urge to vomit; vomiting was the actual forceful expulsion of gastric contents from the mouth.

The secondary outcome variables were severity of nausea, complete response rate, requirements of rescue antiemetics, pain score, opioid PCA consumption, and satisfaction score. A complete response was defined as no reported symptoms of PONV and/or no additional rescue administration of antiemetics.

We recorded nausea severity using a four-point scale: 0, no nausea; 1, mild; 2, moderate; 3, severe. Intravenous metoclopramide was used as the first-line rescue antiemetic drug when two or more episodes of PONV occurred or more than moderate nausea persisted. Pain was assessed using an NRS ranging from 0 (no pain) to 100 (most severe pain) for the three periods. Overall satisfaction was scored using an NRS that ranged from 0 (not satisfied at all) to 100 (no complaints at all), and side effects of the treatment were recorded. Study protocol is available in the supporting information (S1 Text).

### Statistical analysis

We compared the primary and secondary outcomes between the palonosetron and control groups. Chi-square or Fisher’s exact test was used to determine differences in categorical variables, specifically sex, the incidence of nausea and vomiting, requirement for rescue antiemetics, and proportion of complete response. Continuous variables were analyzed with Student’s t-test or the Mann–Whitney U test. Continuous data for the first and second operations in staged bilateral TKA were compared using a paired Student’s *t*-test or Wilcoxon’s signed-rank test.

Sample size was based on the result of a previous study [8]. To detect a 50% reduction from an incidence of 55% in untreated patients, at an alpha level of 0.05 and with a power of 80% using a two-sided test, 112 patients (56 per group) were required. To allow for drop-outs, 60 patients were enrolled in each group. The statistical analyses were conducted using SPSS for Windows software (ver. 19; SPSS Inc., Chicago, IL, USA). P values of <0.05 were considered significant.

## Results

**Fig 1:**
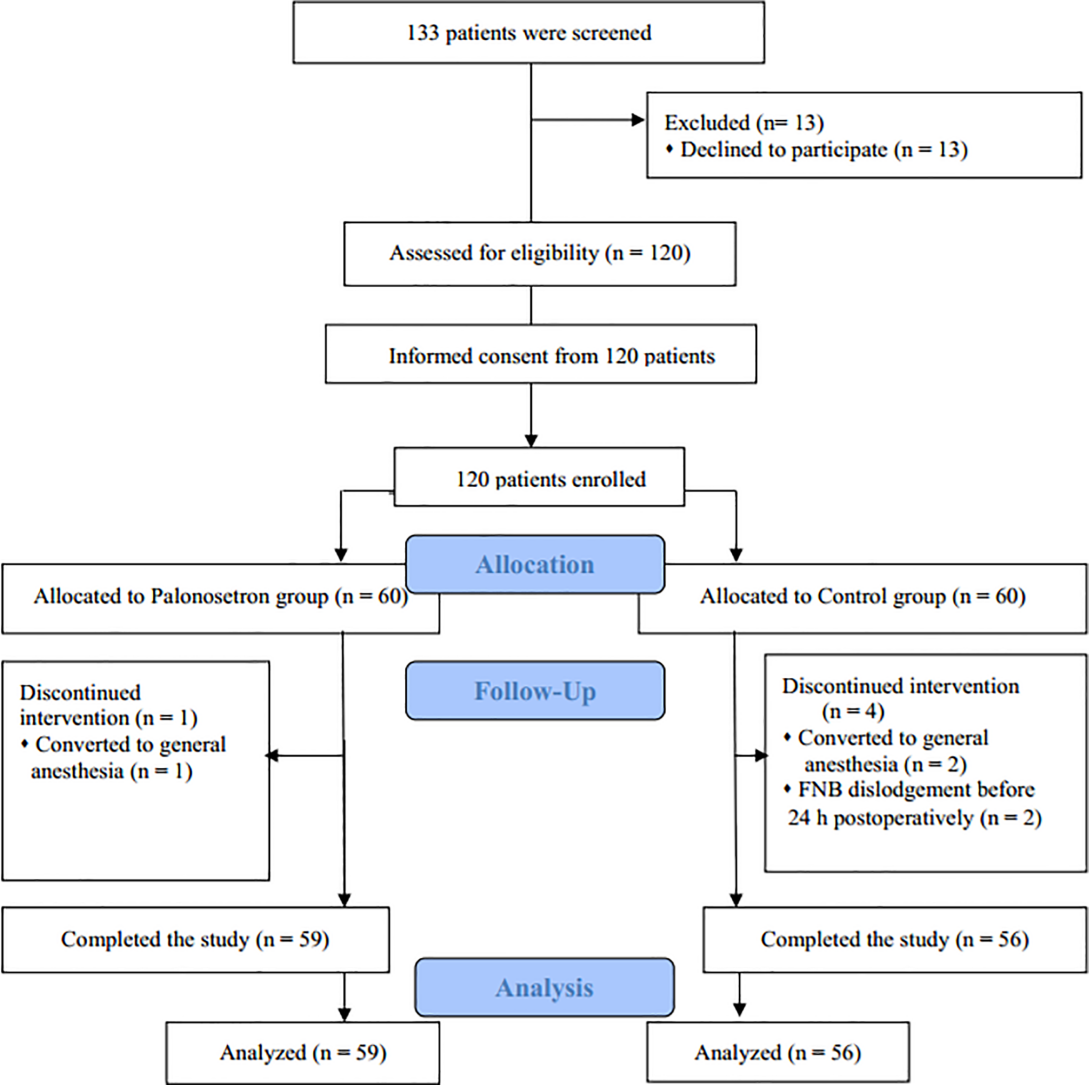
CONSORT flow diagram. FNB, femoral nerve block.

One hundred and twenty high-risk patients were enrolled and 5 patients were excluded from the study. Three patients were excluded due to conversion to general anesthesia and two patients were due to FNB dislodgement (Fig 1). Ultimately, 59 patients in the palonosetron group and 56 in the control group were analyzed. Table 1 summarizes the patient characteristics and information on surgery and anesthesia. Baseline values were comparable between the two groups.

**Table 1:** Patients, operative and anesthetic characteristics.

	Palonosetron (n = 59)	Control (n = 56)	*P* value
Mean age (yr)	72 ± 7	73 ± 6	0.611
Gender (M/F)	0/59	0/56	
Weight (kg)	62 ± 10	62 ± 10	0.837
Height (cm)	152 ± 8	153 ± 7	0.680
Side of surgery(right/left)	31/28	30/26	0.912
Anesthesia time(min)	154 ± 29	147 ± 23	0.177
Height of spinal block (max)	T6 (T5-T10)	T6 (T5-T10)	
Height of spinal block at the end of TKA	T7 (T5-T10)	T8 (T6-T10)	
Intrathecal bupivacaine (mg)	12 ± 1	12 ± 1	0.212
Intraoperative blood loss (ml)	50 ± 10	50 ± 11	

Data are expressed as mean ± SD or numbers (%), or mean (interquartile range).

The overall incidence of PONV during 48 h after surgery was lower in the palonosetron group compared with the controls (22 *vs*. 41%, *p* = 0.028); in particular, at 2–24 h (*p* = 0.026). The incidences of both nausea and vomiting were also lower in the palonosetron group (22 *vs*. 39%, 7 *vs*. 27%, respectively; Table 2). The severity of nausea was lower in the palonosetron group at 2–24 h after surgery (*p* = 0.010; Table 2).

**Table 2:** Incidence and severity of postoperative nausea and vomiting (PONV) during the first 48 h after surgery. Values are number (%).

	Palonosetron (n = 59)	Control (n = 56)	*P* value
**Nausea**			
overall (0–48 h)	13 (22.0) *	22 (39.3)	0.044
0–2 h	3 (5.1)	5 (8.9)	0.483
2–24 h	12 (20.3) *	21 (37.5)	0.042
24–48 h	2 (3.4)	6 (10.7)	0.116
**Vomiting**			
overall (0–48 h)	4 (6.8) *	15 (26.8)	0.007
0–2 h	0 (0)	1 (1.8)	0.487
2–24 h	6 (10.2) *	17 (30.4)	0.007
24–48 h	0 (0)	1 (1.8)	0.487
**PONV**			
overall (0–48 h)	13 (22.0) *	23 (41.1)	0.028
0–2 h	3 (5.1)	5 (8.9)	0.483
2–24 h	12 (20.3) *	22 (39.3)	0.026
24–48 h	2 (3.4)	6 (10.7)	0.116
**Severity of nausea**			
(mild/moderate/severe)			
0–2 h	3 /0 /0	1 /3 /1	0.066
2–24 h	5 /5 /2 *	3 /4 /14	0.010
24–48 h	2 /0 /0	3 /2 /1	0.280

*P < 0.05 between groups.

More patients in the palonosetron group had a complete response (no PONV and no rescue anti-emetics) over the entire postoperative period, notably during 2–24 h (93 *vs*. 73%, Table 3) and the requirement of the rescue antiemetics was lesser during the same period.

**Table 3:** Complete response rates, requirement of rescue antiemetics, and satisfaction score after surgery.

	Palonosetron (n = 59)	Control (n = 56)	*P* value
**Complete response**			
0–2 h	56 (98.3)	49 (92.9)	0.100
2–24 h	47 (93.2) *	35 (73.2)	0.016
24–48 h	57 (96.6)	51 (89.3)	0.214
**Rescue antiemetics**			
0–2 h	1 (1.7)	3 (5.4)	0.356
2–24 h	12 (20.3) *	21 (37.5)	0.042
24–48 h	2 (3.4)	5 (8.9)	0.214
**Satisfaction score (NRS)**			
0–48 h	84 ± 12 *	79 ± 15	0.032

Values are number (%) or mean ± SD.

*P < 0.05 between groups.

The NRS pain scores and PCA consumption were not different, but the overall satisfaction score for postoperative care was significantly higher in the palonosetron group (84 *vs*. 79, *p* = 0.032). Common adverse events related to palonosetron treatment, such as headache, dizziness, or drowsiness, were similar in the two groups.

Forty-seven patients underwent bilateral TKA in a staged operation at a 1-week interval. The incidence of PONV was not different between the first and second operations (Table 4). Patients who underwent a second operation complained of more severe pain and consumed more opioids during 2–48 h postoperatively (Table 5). The satisfaction score according to the order of surgery was comparable. Details of data and CONSORT checklists are available in the supporting information (S1 Data and S2 Text).

**Table 4:** Incidence of emetic events in the first and second operation groups in patients underwent staged bilateral TKA.

	1^st^ operation (n = 47)	2^nd^ operation (n = 47)	*P* value
**Nausea**			
overall (0–48 h)	16 (34.0)	13 (27.7)	0.503
0–2 h	4 (8.5)	4 (8.5)	1.000
2–24 h	16 (34.0)	10 (21.3)	0.167
24–48 h	2 (4.3)	4 (8.5)	0.667
**Vomiting**			
overall (0–48 h)	10 (21.3)	10 (21.3)	1.000
0–2 h	1 (2.1)	2 (4.3)	1.000
2–24 h	10 (21.3)	8 (17.0)	0.600
24–48 h	1 (2.1)	2 (4.3)	1.000

Values are number (%). TKA, total knee arthroplasty.

**Table 5:** Comparisons of pain level, opioid consumption through iv-PCA, and the overall satisfaction level in patients underwent staged bilateral TKA.

	1^st^ operation (n = 47)	2^nd^ operation (n = 47)	*P* value
**Pain score (NRS)**			
0–2 h	2.8 ± 7.7	5.1 ± 1.2	0.272
2–24 h	27.9 ± 10.0 *	35.5 ± 12.8	0.002
24–48 h	30.4 ± 7.5 *	34.7 ± 11.4	0.035
**PCA consumption (ml)**			
0–2 h	0.6 ± 1.2	0.8 ± 1.3	0.566
2–24 h	6.9 ± 8.7 *	10.9 ± 8.2	0.023
24–48 h	19.7 ± 17.9 *	30.0 ± 21.8	0.013
**Satisfaction score (NRS)**			
0–48 h	81 ± 17	77 ± 16	0.214

Values are mean ± SD.

*P < 0.05 between groups. TKA, total knee arthroplasty; NRS, numeric rating scale.

## Discussion

This study demonstrated that a single injection of prophylactic palonosetron was effective in reducing the incidence and severity of PONV while increasing satisfaction score compared with those in the control group, especially 2–24 h after surgery in high-risk patients following TKA. Although more opioids were required for the second operated knee than the first, there was no difference in the incidence of PONV.

Along with the overall incidence of PONV, the incidences of both nausea and vomiting, respectively, were lower in the palonosetron group during the first 48 h postoperatively, notably during the 2–24 h. This concurs with reports that palonosetron is effective at preventing PONV compared with placebo [12,16,17], ondansetron [13,18], dexamethasone [19], and granisetron [18]. Palonosetron, a recently developed second-generation 5-HT_3_ receptor antagonist, has greater binding affinity to the 5-HT_3_ receptor more than 30-fold higher than that of ondansetron or ramosetron [20]. Palonosetron has a specific ternary ring moiety attached to the quinuclidine ring, therefore can bind more tightly to the receptor and multiple palonosetron molecules bind to a single receptor [20]. This allosteric binding and positive cooperativity, which are structurally distinct from the first-generation drugs, lead to effective treatment [21]. Prolonged duration of action of the palonosetron (approximately 40 h, 4-10-fold longer compared to first-generation antagonists) is due to palonosetron-triggered receptor internalization [22]. This greater efficacy has led to potent and long-term antiemetic effect for up to 24 h or more after surgery [23,24].

Our results also showed palonosetron was effective for PONV after the early phase up to 24 h postoperatively. The incidence of nausea was lower in the palonosetron group, especially during 2–24 h postoperatively. Most other studies are in accord with our results, finding that nausea is most prevalent at 2–24 h postoperatively (e.g., 40% incidence with ramosetron and 69.5% with ondansetron during 6–24 h [6], and 35% with palonosetron and 55% with ondansetron during 2–24 h [13]. The incidence of vomiting was also lower in the palonosetron group, particularly during 2–24 h. This result accords well with other reports (e.g., 11% incidence of vomiting in the palonosetron group during 0–24 h [19], 17% with ramosetron during 6–24 h [6], and 10% with palonosetron during 2–24 h ([13]. Palonosetron showed equally effective antinausea and antiemetic actions for PONV during 2–24 h postoperatively in current study.

The complete response rate was noticeably higher in the palonosetron group at 2–24 h postoperatively. Rescue antiemetics use decreased over the exactly same period as the nausea-free period. The severity of nausea increased significantly in the controls at 2–24 h after surgery. Severe nausea was experienced by 70% of the patients who developed nausea in the control group during that period. This severity difference is clinically meaningful and may have affected the patients’ experience and satisfaction.

The incidence of PONV was very low in both groups during the immediate after surgery; 0–2 h postoperatively. Typically, the incidence of PONV is high in the immediate postoperative period [6,8,12,13], however, most of those studies examined patients following general anesthesia [12,13,16–18]. We found a very high complete response rate and an equally low requirement for rescue antiemetics in both groups during the first 2 h after surgery. These positive results may have benefited from the spinal anesthetic residual effects that provide adequate analgesia, thus led to very low pain score and low consumption of PCA. Low opioid consumption prevented the development of opioid-induced PONV. No difference in PONV between the groups in acute postoperative period does not imply that there is no prophylactic effect of palonosetron. Rather, it can be interpreted that due to the specificity of this study; the aftereffects of spinal anesthesia, the antiemetic difference could not be proved under low-emetogenic environment.

No difference was found in PONV incidence at 24–48 h postoperatively between the groups, which may be due to the low incidence in both groups (3 *vs*. 11%). Other studies reported that the incidence of nausea at 24–48 h postoperatively was still very high, at up to 70% in the control group [6,12]. The low PONV in current study may have benefit from appropriate rigorous antiemetic strategy used at our center. Dexamethasone was administered before surgery. Several studies suggest single dose of steroid with palonosetron can provide efficacious antiemetic effect throughout acute and delayed chemotherapy-induced nausea and vomiting [25,26]. Also, in current study, rescue antiemetics during 2–24 h were administered as needed after frequent check-up. This early intervention for optimal therapy may have inhibited the occurrence of delayed PONV by eliminating missed cases without appropriate PONV treatment.

The degree of postoperative pain and use of PCA did not differ between the two groups. Multimodal pain control is used widely and we used identical TKA protocols in both groups. PONV is closely related to effective pain control, because when side effects occur due to PCA use, patients are reluctant to trigger the bolus button and request discontinuation of PCA due to fear of the occurrence of PONV. Therefore, strict pain control is required to accurately compare the effects of antiemetics. Our strict pain control policy underscores the reliability of our study.

We also performed a subgroup analysis of patients who underwent staged bilateral TKA. Patients experienced more severe pain in the second operated knee than in the first at 2–48 h postoperatively. Owing to severe pain, more PCA was used during the same period following the second operation. This is interpreted as a consequence of hyperalgesia, in which pain is detected at a site distant from the injured region and increased [27,28]. This may be partly explained as central sensitization, and therapeutic approaches are required to reduce such hyperalgesia in the course of staged operations [14]. Although more opioids were used following the second operated knee, there was no difference in the incidence of PONV.

Several limitations of this study should be noted. First, our anesthetic and multimodal pain management protocol includes some components that reduce the baseline PONV, such as preoperative dexamethasone, midazolam premedication, continuous propofol infusion, and intraoperative oxygen. These elements may have affected the incidence and severity of PONV; nonetheless, these anesthetic regimens were adopted equally in both groups. Second, assessment of patient satisfaction was subjective. However, we assume that overall patient satisfaction strongly reflects adequate management of two major components of postoperative care: pain and emesis, which could lead to functional impact on daily life. Third, this study was performed under spinal anesthesia, the results cannot be applied directly to PONV under general anesthesia. Fourth, because this study included only female patients, there may be gender bias in behavior of dealing with pain and PONV or scoring them. Fifth, the history of PONV was not thoroughly investigated, which is a strong predictor of PONV. Nevertheless, our results may not be affected by the patient’s previous history, because randomization was used and only high-risk population was included. Finally, unlike palonosetron vs. control confrontation, satisfaction score was not different according to the order of the surgery, 1^st^ vs. 2^nd^ operation. This disparity can be interpreted that it is more important to alleviate PONV than to relieve pain to improve overall patient satisfaction. But since it is not known, further study is required to explain this phenomenon.

In conclusion, a single use of prophylactic palonosetron reduced the incidence and severity of PONV and the need for rescue antiemetics in high-risk patients undergoing TKA with opioid-based postoperative pain management. Palonosetron can be a promising antiemetic agent as part of a comprehensive multimodal pain and PONV protocol to increase patient satisfaction after surgery.

## Supporting information

S1 Text Study protocol_original language and english. (DOCX)

S2 Text CONSORT checklist. (DOC)

S1 Data Study data. (XLSX)
