# Supplementary material for: Effects of palonosetron for prophylaxis of postoperative nausea and vomiting in high-risk patients undergoing total knee arthroplasty: A prospective, randomized, double-blind, placebo-controlled study
Source: PLoS One. 2018 May 14;13(5):e0196388. doi: 10.1371/journal.pone.0196388 (PMC5951557; doi:10.1371/journal.pone.0196388)
Supplement: S1 Text — (DOCX) [file pone.0196388.s001.docx]

**Study Protocol**

| **Effects of palonosetron for prophylaxis of postoperative nausea and vomiting in high-risk patients undergoing total knee arthroplasty** |
| --- |

Seoul National University Bundang Hospital

Hye-Min Sohn

1. Study name and Type

Effects of palonosetron for prophylaxis of postoperative nausea and vomiting in high-risk patients undergoing total knee arthroplasty

A prospective, randomized, double-blind, placebo-controlled study

1. Organization and Address

Seoul National University Bundang Hospital

300 Gumi-dong, Bundang-gu, Seongnam-si, Gyeonggi-do

1. Research Team

Hye-Min Sohn, Young-Tae Jeon, Jung-Hee Ryu, Jae-Sung Lee,

SMG-SNU: Jin-Young Hwang

Orthopedic surgery: Jang Jong-Beom,

1. Study Backgrounds

Incidence of postoperative nausea and vomiting (PONV) in patients undergoing arthroplasty is very high from 68% to 83% in patients without prophylactic antiemetics. Multimodal pain protocols to reduce pain after total knee arthroplasty (TKA) provokes higher emetic events than traditional pain control. Various methods are recommended to decrease PONV; prophylactic antiemetics, regional anesthesia, use of propofol, oxygen and fluid infusion, a minimum use of opioids, use of dexamethasone. Despite uses of these preventive methods, still high incidence of PONV (40%) is reported in relation to TKA.

Palonosetron, the latest 5-HT_3_ receptor antagonist, has longer half-life and high receptor-affinity than other antagonist. Also it is known to be effective against nausea and vomiting in patients using anticancer drugs or used as preventive agents against PONV. However, the antiemetic efficacy achieved by palonosetron prophylaxis in high risk patients remains unclear.

Pain is increased in patients undergoing staged TKA, in whom the second operated knee had greater sensitivity as a result of surgical injury to the first operated knee and more analgesics are required. The effect of antiemetics on the PONV was not evaluated in patients undergoing the second surgery of a staged bilateral TKA.

**Aims:** The overall aim of this prospective, randomized, double-blinded trial was to investigate the prophylactic antiemetic efficacy of palonosetron in high-risk patients with multimodal analgesics including fentanyl-based patient controlled analgesia (PCA) following TKA.

**Primary objective:** The primary objective of the trial is to determine whether prophylactic palonosetron in patients undergoing TKA decrease PONV during 48 h after surgery. Specifically, we compared the incidence of individual nausea, vomiting, and overall PONV with that of control groups, at three time periods: 0-2 h, 2-24 h and 24-48 h after surgery.

**Secondary objective:** we investigated the influence of palonosetron prophylaxis on the postoperative pain level and opioid consumption. Additionally, we performed sub-group analysis; comparing the emesis and pain in patients undergoing bilateral staged TKA according to the order of surgery.

1. Code name of the study drug, formulation

Code name: PLNS7I, formulation: 0.075 mg vial

1. Participants characteristics

Female patients who undergo elective total knee arthroplasty because of degenerative arthritis under spinal anesthesia

1. Expected Research Duration

October 1, 2011 - September 30, 2012

1. Methods/Design
2. Summary

We randomly assigned the patients into 2 groups, administering palonosetron 0.075 mg or no prophylaxis (control group, same volume (1.5 ml) of 0.9% normal saline). In patients undergoing both knee arthroplasty, there was one-week interval between the two surgeries.

All patients were given the same anesthetics and multimodal pain protocol, except palonosetron. On arrival at the surgical reception area, patients were premedicated with midazolam 0.03 mg/kg intravenously and a continuous FNB was established with ultrasound-guided technique. Spinal anesthesia consisted of bupivacaine 10 to 15 mg and adjuvant fentanyl 15 to 20 μg. Palonosetron were injected just before connection to iv-PCA in palonosetron group.

1. Study Participants

**Inclusion criteria:**

aged 18-85 years female patients

scheduled for unilateral or staged bilateral TKA for primary osteoarthritis under spinal anesthesia

postoperative pain management using intravenous PCA

continuous femoral nerve block (FNB)

ASA physical status I or II

**Exclusion criteria:**

patients undergoing revision TKA

simultaneous bilateral TKA

general or epidural anesthesia

patients who took opioids or steroids within 1 week of surgery

antiemetic medication within 1 day of surgery

renal insufficiency (serum Cr > 1.6 mg/dl)

unable to use intravenous PCA or continuous FNB

unable to understand the numeric rating scale (NRS) for pain or express the degree of PONV

abused alcohol or drugs

comorbidities of the digestive system

1. Sample size calculation

Sample size was based on the result of a previous study (7). To detect a 50% reduction from an incidence of 55% in untreated patients, at an alpha level of 0.05 and with a power of 80% using a two-sided test, 112 patients (56 per group) were required. To allow for drop-outs, 60 patients were enrolled in each group.

1. Control group

Patients who did not receive palonosetron prophylaxis following total knee arthroplasty were treated as a control group. They received placebo- same volume of saline.

1. Randomization

Block randomization with a block size of four was used based on a computer-generated schedule. The group assignments were sealed in sequentially numbered, opaque envelopes. Patients undergoing unilateral TKA were assigned to either the palonosetron or control group. Patients undergoing staged bilateral TKA, with a 1-week interval between stages 1 and 2, were assigned to one group randomly for the first knee and then to the other group automatically for the second knee.

1. Blinding

The patients and anesthesia providers were blinded to the group assignments; a trained nurse who was not involved in the study prepared the drugs in identical syringes. Another anesthesiologist blinded to the group allocations evaluated the patients 0–2, 2–24 and 24–48 h after surgery for the outcome measures.

1. Intervention arm (Test drug dose, method of administration, duration of administration)

Thirty minutes before the end of surgery, either palonosetron (0.075 mg, palonosetron group, n = 60) or saline (control group, n = 60) was injected according to the group assignment, and intravenous PCA was started in succession.

1. Control arm

Thirty minutes before the end of surgery, 1.5 ml of normal saline (control group, no prophylaxis group) was injected according to the group assignment just before the start of intravenous PCA.

1. Combination therapy

Not applicable

1. Observation measurements:

During 3 periods; 0–2, 2–24 and 24–48 h after surgery

Incidence and severity of nausea,

Vomiting

Pain scores: Numeric rating scale, NRS

Requirements of rescue antiemetics, analgesics

PCA consumption

Other side effects (headache, dizziness etc.)

1. Compliance assessment

Not applicable

1. Outcome measures:

During 3 periods; 0–2, 2–24 and 24–48 h after surgery

Incidence and severity of nausea, vomiting and overall PONV

Pain scores: Numeric rating scale, NRS

Requirements of rescue antiemetics, analgesics

Complete response

Intravenous PCA consumption

Other side effects (headache, dizziness etc.)

Satisfaction score (NRS)

1. Side effects and reporting methods

Anesthesia methods and pain control methods are commonly used standard clinical practice, and palonosetron has been reported to be safely used. Side effects such as headache, drowsiness, dizziness, etc., may occur during the study periods and investigators and medical staffs should pay careful attention.

1. Statistical analysis

We compared the primary and secondary outcomes between the palonosetron and control groups. Chi-square or Fisher’s exact test was used to determine differences in categorical variables, specifically sex, the incidence of nausea and vomiting, requirement for rescue antiemetics, and proportion of complete response. Continuous variables were analyzed with Student’s t-test or the Mann–Whitney U test. Continuous data for the first and second operations in staged bilateral TKA were compared using a paired Student’s *t*-test or Wilcoxon’s signed-rank test. The statistical analyses were conducted using SPSS for Windows software (ver. 19; SPSS Inc., Chicago, IL, USA). P values of <0.05 were considered significant.

1. Safety considerations for subjects

**Informed consent**

This study was approved by the institutional review board of Seoul National University Bundang Hospital and registered at the Clinical Research Information Service (cris.nih.go.kr). We studied 120 patients undergoing elective TKA after obtaining written informed consents before recruiting.

Both control and intervention arms will be given the same information about the trial

1. Study timescale

|  | After approval of IRB (month) | | | | | | | | | | | |
| --- | --- | --- | --- | --- | --- | --- | --- | --- | --- | --- | --- | --- |
|  | 1 | 2 | 3 | 4 | 5 | 6 | 7 | 8 | 9 | 10 | 11 | 12 |
| Research, Plan the protocol | 🡨--🡪 | |  |  |  |  |  |  |  |  |  |  |
| Patient recruitment |  | 🡨---------------------🡪 | | | | |  |  |  |  |  |  |
| Data collection and analysis |  | 🡨---------------------🡪 | | | | |  |  |  |  |  |  |
| Statistical analysis, |  |  |  |  |  |  |  |  | 🡨-----🡪 | | |  |
| Data cleaning Writing-up |  |  |  |  |  |  |  |  | 🡨-------🡪 | | | |

1. Ethics

Anonymity is the principal ethical challenge. To address this, participants will be given a unique ID number and pseudonym. Identifiable names of people, places will be removed from each transcript. Digital recordings will be held on a dedicated secure University server, and will only be accessible to those directly involved in the study. All quotations in reports, publications, and presentations will be presented in an anonymous format.

It was conducted according to the revised Declaration of Helsinki of the World Medical Association and ICH GCP guidelines for good clinical trial practice.

1. Reference

1) Apfel, C. C.; Laara, E.; Koivuranta, M.; Greim, C. A.; and Roewer, N.: A simplified risk score for predicting postoperative nausea and vomiting: conclusions from cross-validations between two centers. *Anesthesiology,* 91: 693-700, 1999.

2) Gan, T. J.: Postoperative nausea and vomiting--can it be eliminated? *JAMA,* 287: 1233-6, 2002.

3) Wulf, H.; Biscoping, J.; Beland, B.; Bachmann-Mennenga, B.; and Motsch, J.: Ropivacaine epidural anesthesia and analgesia versus general anesthesia and intravenous patient-controlled analgesia with morphine in the perioperative management of hip replacement. Ropivacaine Hip Replacement Multicenter Study Group. *Anesth Analg,* 89: 111-6, 1999.

**연구계획서**

| **슬관절 전치환술을 받는 고위험군 환자에서**  **palonosetron의 수술 후 오심 구토에 관한 영향** |
| --- |

**분당서울대학교병원**

**손 혜 민**

1. 연구의 명칭 및 단계

슬관절 전치환술을 받는 환자에서 palonosetron의 수술 후 오심 구토에 관한 영향

학술연구, 전향적, 무작위배정

1. 실시 기관명 및 주소

경기도 성남시 분당구 구미동 300 분당서울대학교병원

1. 연구책임자 및 담당자

연구책임자: 마취통증의학과 손혜민

공동연구자: 마취통증의학과 전영태, 유정희, 이재성,

정형외과 장종범

보라매병원 황진영

1. 연구의 목적 및 배경

관절 수술을 받는 환자에서 수술 후 오심, 구토(Postoperative nausea and vomiting, PONV)는 아주 흔하여 예방적 전처치가 없는 경우 빈도가 68에서 83%까지 이른다고 보고되어 있다. PONV의 위험인자로는 여성, 비흡연, 과거 수술 후 오심, 구토나 멀미의 기왕력, 수술 후 아편유사제의 사용등이 알려져 있으며 이들 위험 인자 중 두개 이상에 해당하는 고위험군에서는 예방적 항구토제의 사용이 권장되고 있다. 슬관절 전치환술을 시행받는 환자들은 대부분이 비흡연 여성이고 수술 후 아편유사제를 진통 목적으로 사용하고 있어서 거의 모든 환자가 수술 후 오심, 구토의 위험인자를 가진 고위험군에 해당한다고 볼 수 있다. PONV를 줄이기 위해 예방적 항구토제의 사용, 부위마취, propofol의 사용, 충분한 산소와 수액 공급, 최소한의 아편유사제 사용, dexamethasone의 사용등이 권장되고 있다. 본 기관에서 슬관절 전치환술을 받는 환자에서 이러한 예방적 방법들을 사용하고 있음에도 50% 이상의 환자에서 PONV가 발생하고 있다. 특히 예방적 항구토제로 사용되고 있는 ramosetron은 약제 비용이 1 ample당 4만원으로 고가의 약제여서 환자들에게 부담이 되고 있다. 최근에 ramosetron과 비슷한 작용 시간과 효능을 가지면서 가격은 저렴한 palonosetron이 임상에 사용되고 있는데 아직까지 슬관절 전치환술을 받는 환자에서 그 효과가 증명된 바가 없어 이에 대한 연구가 필요하다. 현재 본 기관에서 상용되는 다중진통 protocol은 수술 후 진통에 효과적이나 아편유사제가 많이 사용되어 다른 수술에 비해 오심, 구토의 발생 빈도가 높아서 palonosetron이 효과적이라면 환자의 만족도 증가에 많은 도움이 될 것이다.

1. 연구약의 코드명, 제형

코드명:PLNS7I 제형: 0.075 mg vial

1. 대상질환

퇴행성 관절염으로 슬관절 전치환술을 받는 여성 환자

1. 예상연구기간

2011년 10월 1일- 2012년 9월 30일

1. 연구방법
2. 연구방법 개요

무작위 배정표를 이용하여 두 군으로 나누어서 무작위 배정을 시행한다. 일측만 수술을 받는 환자는 무작위 배정표에 의해서 palonosetron 0.075mg 투여 군과 no prophylaxis 군으로 배정한다. 1주 간격으로 수술을 받는 환자는 첫번째 무릎을 기준으로 Prophylaxis 군과 no prophylaxis 군으로 나누고 두 번째 무릎은 첫 번째 무릎과 반대 방법으로 시행한다. 대퇴신경차단술을 시행한 후 척추마취를 시행하고 수술이 끝난 후 intravenous patient-controlled analgesia (IV-PCA)로 통증 조절을 한다. IV-PCA 투여 전 prophylaxis군에서 palonosetron 0.075mg을 정주한다.

1. 피험자의 선정, 제외 기준

**선정기준:**

18-85 세 여성

척추마취 하에 퇴행성 관절염으로 일측성 슬관절 전치환술을 시행

수술 후 iv PCA 로 통증조절을 하는 환자

지속적인 FNB 로 통증조절을 하는 환자

ASA 1,2 에 해당하는 환자

연구 내용을 이해하고 연구 참여에 서명 동의서를 제공하는 환자를 대상으로 한다.

**제외기준:**

부위마취의 금기증으로 부위마취를 시행하지 못한 경우,

부위마취가 실패하여 전신마취가 시행된 경우,

IV-PCA나 대퇴신경 차단이 중단된 경우,

steroid therapy를 받고 있는 경우,

수술 중 propofol을 사용하지 못한 경우는 제외한다.

1. 목표 피험자의 수 및 산출 근거

일측성 슬관절 전치환술을 시행 받는 대다수 환자의 Apfel’s risk factor는 3개 이상으로 평균 PONV risk(control event rate)는 약 60%이며, 본 regimen의 relative reduction rate을 50%로 가정할 때, 2-sided chi-square test를 이용하여 30% PONV incidence reduction(각각 30%의 absolute reduction rate와 treatment event rate)을 alpha 0.05, 80% power로 detection 할 수 있는 sample size는 각 군당 49으로 중도 탈락으로 배제되는 환자를 감안하여 각 군당 60명, 총 120명의 일측성 인공관절 치환술 환자를 대상으로 한다.

1. 비교군 설정

척추마취하에 슬관절 전치환술 수술 시 palonosetron prophylaxis를 받지 않는 환자를 대조군으로 하고 palonosetron prophylaxis 받는 환자를 비교군으로 설정한다.

1. 무작위배정

무작위 배정표를 이용하여 두 군으로 나누어서 무작위 배정을 시행한다. 일측만 수술을 받는 환자는 무작위 배정표에 의해서 palonosetron prophylaxis 시행 군과 시행하지 않는 군으로 배정한다. 1주 간격으로 수술을 받는 환자는 첫 번째 무릎을 기준으로 Prophylaxis 군과 No Prophylaxis 군으로 나누고 두 번째 무릎은 첫 번째 무릎과 반대 방법으로 시행한다. 양측 동시에 시행 받는 환자는 prophylaxis를 시행하지 않는 군으로 배정한다.

1. 눈가림법 적용

피험자를 올바르게 관리하기 위해 봉투에 일련번호가 매겨져 있게 하였으며 무작위배정표는 연구자(기록자)에게 공개되지 않게 한다. 2명의 연구자가 참여하여 눈가림되지 않은 한 명의 연구자가 무작위 군 배정에 따라 palonosetron prophylaxis를 시행하거나 시행하지 않게 된다. 눈가림된 다른 한 명의 연구자가 관찰항목 (술후 오심 구토 여부, 진토제 투여, 술후 통증, 환자만족도)을 기록한다.

1. 시험약 투여량, 투여방법, 투여 기간 및 설정 사유

무작위 배정에 의해 지정된 prophylaxis 군에 수술 후에 IV-PCA 적용 시 palonosetron 0.075mg을 투여하고 no prophylaxis군에는 예방적 항구토제를 투여하지 않는다.

1. 대조약 사용 시 그 선택 사유

해당사항 없음,

1. 병용요법

해당사항 없음

1. 관찰항목, 관찰 검사 방법 및 임상검사항목

수술 후에 2시간, 24시간, 48시간에

메스꺼움 여부와 정도(nausea, nausea severity)

울렁거림(retching)

구토(vomiting)

통증정도(Visual analogue scale, VAS)

Antiemetics, analgesics 요구량

PCA 주입량

다른 side effect(headache, dizziness등) 여부를 관찰한다

1. 순응도 평가

해당사항 없음

1. 효과평가 변수, 평가방법 및 해석 방법
   - - 1. Nausea의 발생 빈도 및 정도(severity): 수술 후 0~2 시간, 2~24, 24~48 시간에서 각각의 측정 기간 동안 nausea의 발생 빈도 및 11-point numerical VAS를 이용한 severity 정도
       2. Vomiting의 발생 빈도 및 시간: 수술 후 0~2 시간, 2~24 시간, 24~48 시간에서 각각의 측정 기간 동안 1회 이상 구토(vomiting)의 발생 빈도 및 발생 시간
       3. Rescue antiemetics의 빈도: 수술 후 0~2 시간, 2~24 시간, 24~48 시간에서 각각의 측정 기간 동안 rescue antiemetics가 투여된 빈도
       4. Complete response: 수술 후 0~2 시간, 2~24 시간, 24~48 시간에서 각각의 측정 기간 동안 no PONV & no rescue antiemesis의 빈도
       5. VAS를 이용한 통증 수준: 수술 후 0~2 시간, 2~24 시간, 24~48 시간에서 각각의 측정 기간 동안 통증 수준
       6. IV-PCA를 통한 진통제 사용량: 수술 후 0~2 시간, 2~24 시간, 24~48 시간에서 각각의 측정 기간 동안 사용된 PCA 사용량
       7. VAS를 이용한 PONV management에 관S한 환자 만족도: 수술 후 48시간째
2. 안정성 평가기준, 평가 방법 및 보고 방법

마취방법과 통증 조절 방법은 임상에서 통상적으로 이루어지고 있는 방법이고 palonosetron에 대해서도 안정성은 보고되어 있어 본 연구에서 위험성과 부작용 발생 가능성이 높지는 않다. 발생할 수 있는 부작용으로 headache, drowsiness, dizziness 등이 있어 PONV 측정 기간 동안 이 증상들의 발생 빈도를 조사한다.

1. 통계 분석 원칙 및 방법

두 군의 비교를 위한 주 종속변수(primary outcome variable)은 PONV incidence, nausea severity, rescue antiemesis incidence, complete response로 설정한다. 그 외에 통증 수준, PCA 사용량, 환자의 만족도, 부작용 발생 빈도를 부 종속변수 (secondary outcome variable)로 설정한다. 두 군의 비교에서 continuous numerical data는 student t-test를 사용하고, categorical variable인 경우에는 chi-square 방법을 사용한다. 통계분석은 SPSS (version 15.0, Chicago, USA)를 이용하고 통계적 유의성은 p < 0.05로 설정한다.

1. 피험자에 대한 안전성의 배려

시술과 연구는 마취통증의학과 전문의에 의해 이루어지도록 한다. 환자의 안전 상태를 지속적으로 감시하고 합병증 발생 시 즉각적으로 대처한다.

1. 연구 수행 일정표

| 연 구 내 용 | 추 진 일 정 (2011. 10.1 ~ 2012. 9. 30, 12개월) | | | | | | | | | | | |
| --- | --- | --- | --- | --- | --- | --- | --- | --- | --- | --- | --- | --- |
|  | 1 | 2 | 3 | 4 | 5 | 6 | 7 | 8 | 9 | 10 | 11 | 12 |
| 참고문헌 고찰,실험계획 | 🡨--🡪 | |  |  |  |  |  |  |  |  |  |  |
| Data 수집 및 분석 |  | 🡨---------------------🡪 | | | | | | | |  |  |  |
| 추가실험 |  |  |  |  |  |  |  |  | 🡨-----🡪 | | |  |
| 자료 정리 및 논문작성 |  |  |  |  |  |  |  |  | 🡨-------🡪 | | | |

1. 연구의 윤리성 확보를 위한 방안

연구 윤리를 위하여 헬싱키 선언을 준수하고 환자의 건강과 권리가 보호되도록 한다. 통상적으로 사용되는 마취 방법과 다르지 않고 항구토를 위하여 사용되는 약제의 종류만 변화시켜 환자에게 어떠한 위해가 가지 않도록 한다.

1. 참고문헌

1) Apfel, C. C.; Laara, E.; Koivuranta, M.; Greim, C. A.; and Roewer, N.: A simplified risk score for predicting postoperative nausea and vomiting: conclusions from cross-validations between two centers. *Anesthesiology,* 91: 693-700, 1999.

2) Gan, T. J.: Postoperative nausea and vomiting--can it be eliminated? *JAMA,* 287: 1233-6, 2002.

3)Wulf, H.; Biscoping, J.; Beland, B.; Bachmann-Mennenga, B.; and Motsch, J.: Ropivacaine epidural anesthesia and analgesia versus general anesthesia and intravenous patient-controlled analgesia with morphine in the perioperative management of hip replacement. Ropivacaine Hip Replacement Multicenter Study Group. *Anesth Analg,* 89: 111-6, 1999.
